# Three dimension printing talar prostheses for total replacement in talar necrosis and collapse

**DOI:** 10.1007/s00264-021-04992-9

**Published:** 2021-03-05

**Authors:** Mi duo Mu, Qian dong Yang, Wan Chen, Xu Tao, Cheng ke Zhang, Xuan Zhang, Mei ming Xie, Kang lai Tang

**Affiliations:** 1 The First Affiliated Hospital of Military Medical University of the Army, Chongqing, China; 2 The General Hospital of Western Theater Command, Chengdu, China

**Keywords:** Talus necrosis, 3D printing technology, Artificial talus

## Abstract

**Background:**

Reconstructing bone structures and stabilizing adjacent joints are clinical challenges in treating talar necrosis and collapse (TNC). 3D printing technology has been demonstrated to improve the accuracy of talar replacement. This study aimed to evaluate anatomical talar replacement and the clinical results.

**Methods:**

Nine patients with TNC were enrolled between 2016 and 2020. The prosthetic shape and size were designed by CT post-processing and mirror symmetry technology. The clinical outcomes included radiographic parameters of the forefoot, hindfoot, and ankle alignment, ankle activity, recurrent pain, and peri-operative complications.

**Results:**

After a mean follow-up of 23.17 ± 6.65 months, degenerative arthritis and prosthetic dislocation and other complications were not observed on plain radiographs. Each 3D-printed talar prosthesis was placed in the original anatomical position. The parameters which have significant changes pre-operative and post-operative are as follows: talar height, 27.59 ± 5.99 mm and 34.56 ± 3.54 mm (95% CI − 13.05 to − 0.87, *t* = 2.94, *P* = 0.032) and Meary’s angle, 11.73 ± 4.79° and 4.45 ± 1.82° (95% CI 1.29~22.44, *t* = 2.89, *P* = 0.034). The AOFAS hindfoot score improved from 26.33 ± 6.62 to 79.67 ± 3.14 at the final follow-up (95% CI 43.36~63.30, *t* = 13.75, *P* = 0.000). The VAS score decreased from 6.33 ± 1.03 to 0.83 ± 0.75 (95% CI 4.40~6.60, *t* = 12.84, *P* = 0.000). The post-operative satisfaction scores regarding pain relief, activities of daily living, and return to recreational activities were good to excellent, and the change of activity range was statistically significant.

**Conclusions:**

The 3D printing patient-specific total talar prostheses allowed anatomical reconstruction in TNC. This novel treatment with 3D-printed prostheses could serve as a reliable patient-specific alternative in TNC.

## Introduction

Talar necrosis and collapse (TNC) is caused by avascular necrosis of the talus [1]. Irregular positioning or dislocation of talus, incomplete correction of pathological bone condition, and instability of four adjacent articular facet are related factors of talus functional reconstruction. Reconstructing talar anatomy is a challenge in recovering biological function of ankle. Various methods have been reported, including bone grafting and ankle arthrodesis [2–6]. There has been controversy about the ideal method for reconstruction in talar necrosis and collapse and whether performing an intervention during one of the 4 phases in particular would lead to optimal biological recovery and long-term outcomes. Autologous bone can be used to fill bone defects, but the disadvantage is that the survival rate is not desirable [4] and it cannot replicate the original talar shape. While ankle arthrodesis can lead to a higher rate of bone union, it will cause the loss of ankle function [4, 7].

Although the relevant surgical techniques and talar prostheses have improved, full reconstruction of the talus remains a major clinical challenge [8–12]. 3D printing technology has been widely used in clinical practice [13–16], which is more suitable for manufacturing small batches of products with complex shapes and structures. Therefore, 3D printing technology shows strong promise for surgeons to provide patients with efficient, accurate, and personalized medical solutions.

The purpose of the study was to evaluate 3D printing as a new method for talar replacement in TNC. We hypothesized that 3D printing the talar prosthesis can lead to accurate positioning of the prosthesis and promote fusion of the prosthesis and bone at the interface.

## Materials and methods

### Patients

We reviewed the clinical and imaging data of patients with TNC who were treated with 3D printing total talar replacement from 2016 to 2020 by a senior author with extensive foot surgery experience.

Inclusion criteria were as follows: (1) lack of efficacy with conservative treatments, including oral drugs and physical rehabilitation for at least 12 months; (2) severe hindfoot pain, instability, and TNC diagnosed by symptoms and findings on X-ray, CT, or MRI examination; (3) a follow-up period of at least 1 year; and (4) cannot be solved by other traditional surgical methods

Exclusion criteria were as follows: (1) age less than 18 years, (2) active osteomyelitis, (3) inability to tolerate anesthesia and surgery, (4) severe osteoporosis, and (5) hindfoot deformity requiring other surgery.

All patients met the inclusion and exclusion criteria: the mean age was 38.33 ± 16.19 years old (range, 22~65 years), and the mean duration of symptoms was 29.66 ± 33.99 weeks (range, 6~96 weeks). Five patients underwent surgery on the left foot, and 4 on the right; nine patients suffered end-stage TNC and presented with pain surrounding the ankle, difficulty walking, and local pain aggravation with swelling (Table 1). Pre-operative MRI and CT both indicated complete TNC and an intact foot. These patients requested surgical treatment with the preservation of ankle joint function.

**Table 1:** Patient characteristics (*n* = 9)

Characteristic	Values
Mean age, years	38.3 (22~65)
Mean BMI, kg/m^2^	24.8 (19.1~32.3)
Caprini thrombosis risk assessment scale	0.22 (0~2)
Smokers	2 (22%)
Diabetics	0
Prior surgery on ankle	0
Post-operative complications	0

This study was approved by the institutional review board ethics committee of the Southwest Hospital Affiliated with the Army Medical University (No. ECFAH2006051), and written informed consent was obtained from the participants.

### Pre-operative evaluation and planning

**Fig. 1:**
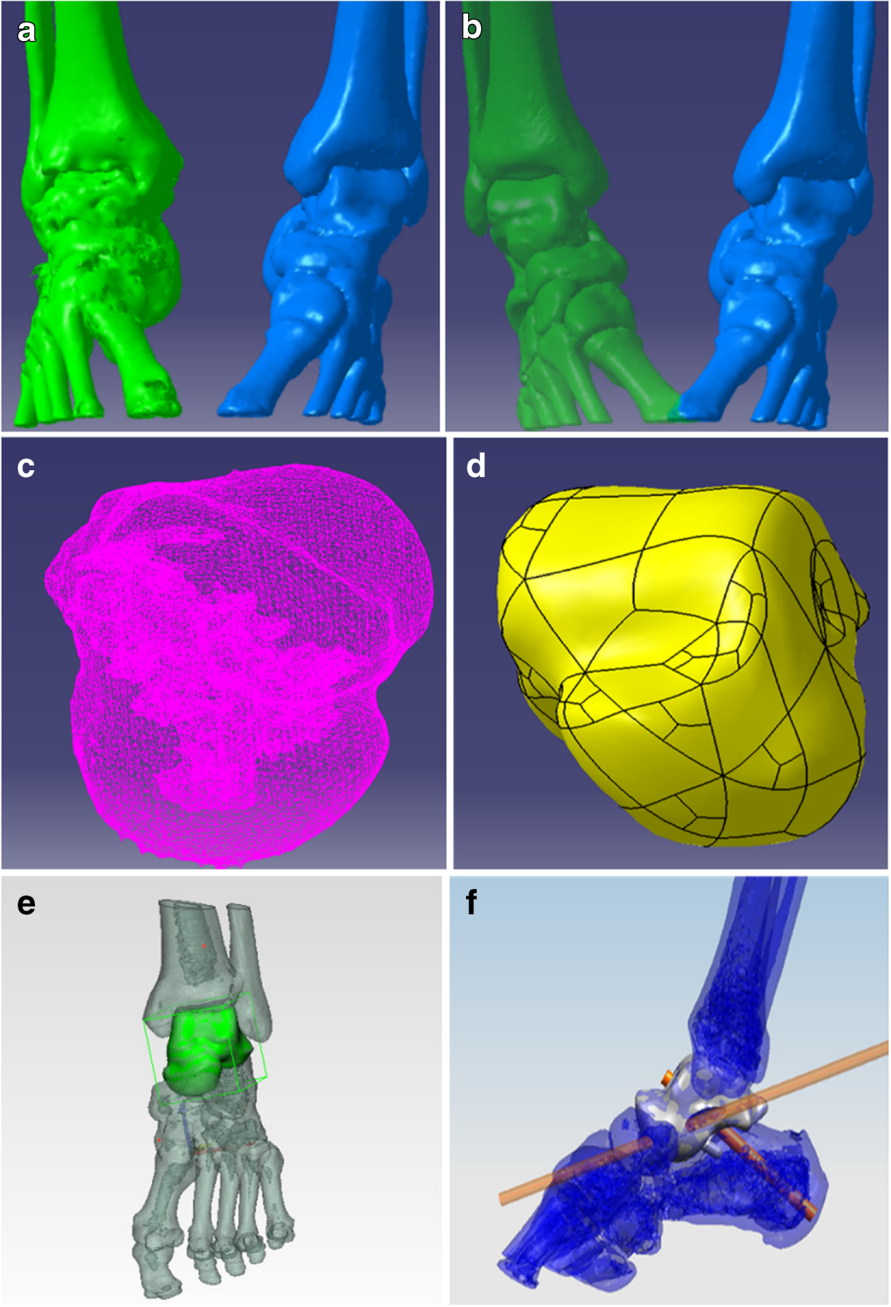
Individualized 3D modeling of talar prosthesis. **a** The data of the affected talus were acquired from the right foot, and the intact data were obtained from the intact foot. **b** The raw data for surgical reconstruction with the individualized talus were acquired by symmetrization and registration. C.Raw data for talar modeling were obtained with reverse repair technology. **d** The 3D talar prosthetic model was simulated. High-precision polishing and screw placement were included in the design of the prosthetic tibiotalar articular facet. **e** Determination of the locating column of the talar prosthesis. **f** The cannulated screw channel for fixation of the talonavicular and subtalar joints was stimulated

Complete data of the affected area were acquired by CT image processing and segmentation using 3D CT postprocessing technology (Fig. 1a), and the intact 3D raw data of the affected side were obtained by reconstruction and matching based on mirror and data registration technology (Fig. 1b). Serious defects in the data of the necrotic talus were repaired using reverse repair technology to provide nondefective raw data for talar reconstruction. Then, the tibiotalar, talonavicular, and subtalar articular facets were analyzed and processed (Fig. 1c); finally, the accurate 3D reconstruction of the talar prosthesis was finished (Fig. 1d).

The talar prosthetic model was fitted to match the adjacent joints(the configuration of the adjacent bones of the upper talus of the affected foot is an accurate mirror image of the complete opposite side), and the locating column of the talar prosthesis was determined (Fig. 1e). Then, the screw fixation channel was established, the porous talonavicular and subtalar articular structures were designed, and the virtual surgical simulation of total talar prosthetic implantation and fixation was completed (Fig. 1f).

**Fig. 2:**
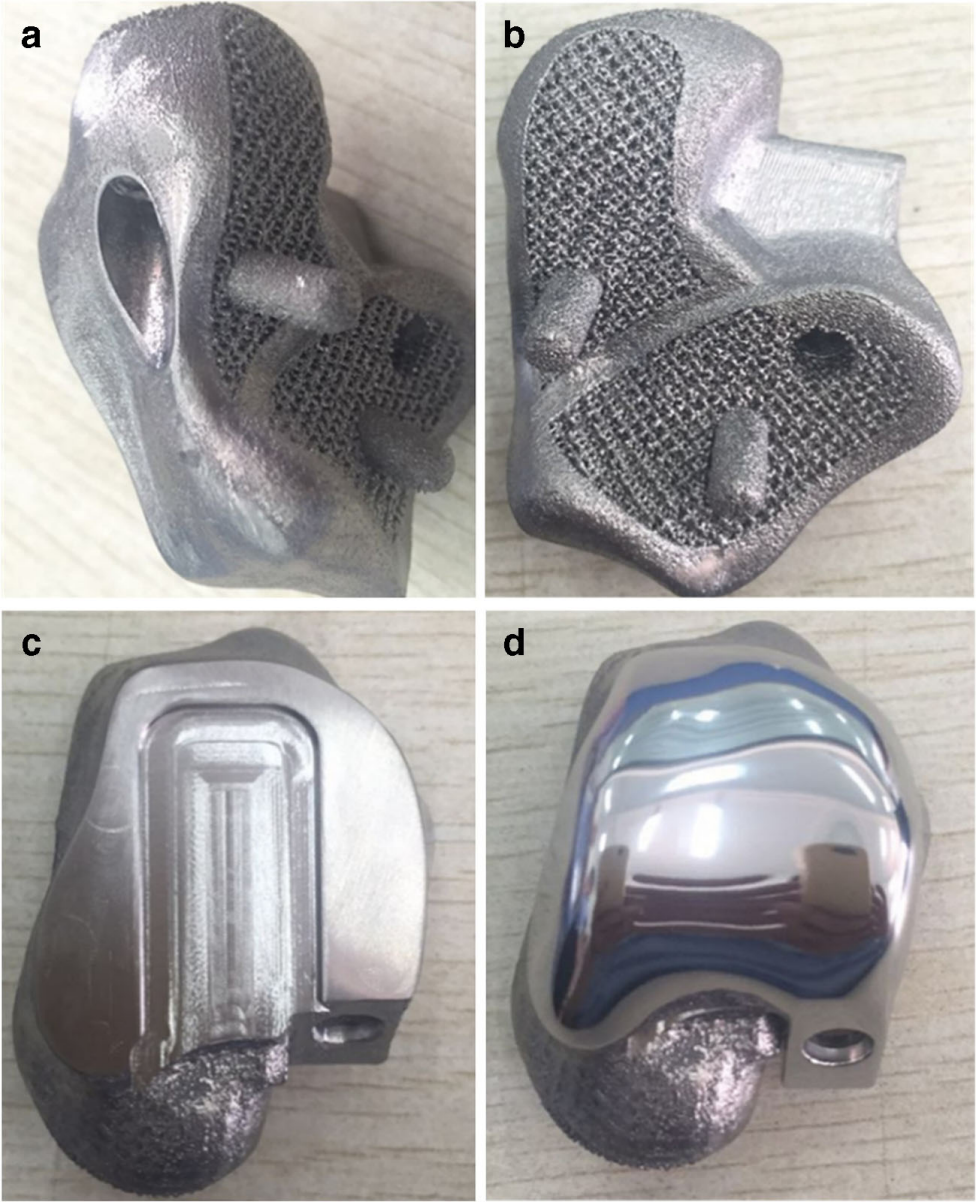
**a**, **b** Processing of porous talonavicular and subtalar articular structures and establishment of the fixation screw channel. **c** High-precision dovetail slot design of prosthetic tibiotalar articular facet. **d** Bright polishing of articular facet after assembly of the tibiotalar prosthesis

The locating column of the talar prosthesis at the calcaneal side and the position of the cannulated screws for fixation of the talonavicular and subtalar joints were determined, and then, the talar prosthesis was located after drilling according to the test model. The 3D-printed structure was made porous on the sides of the talonavicular and subtalar joints (Fig. 2a and b). 3D printing was completed using the Arcam EBM Q10 system (USA). The specific casting process includes mirror polishing of tibial articular surface, polishing and trimming of talus matrix, ultrasonic cleaning, fine cleaning, and drying. Finally, the articular surfaces of talus and matrix are assembled and confirmed in the purification workshop, and then packaged after sterilization. Titanium alloy powder was used as the talar structure material, and cobalt-chromium-molybdenum alloy powder was used as the articular facet material. The high-precision dovetail slot design and screw channel fixation of the prosthetic tibiotalar articular facet were completed after assembly (Fig. 2c). The articular facet was subjected to bright polishing (Fig. 2d).

### Operative technique

**Fig. 3:**
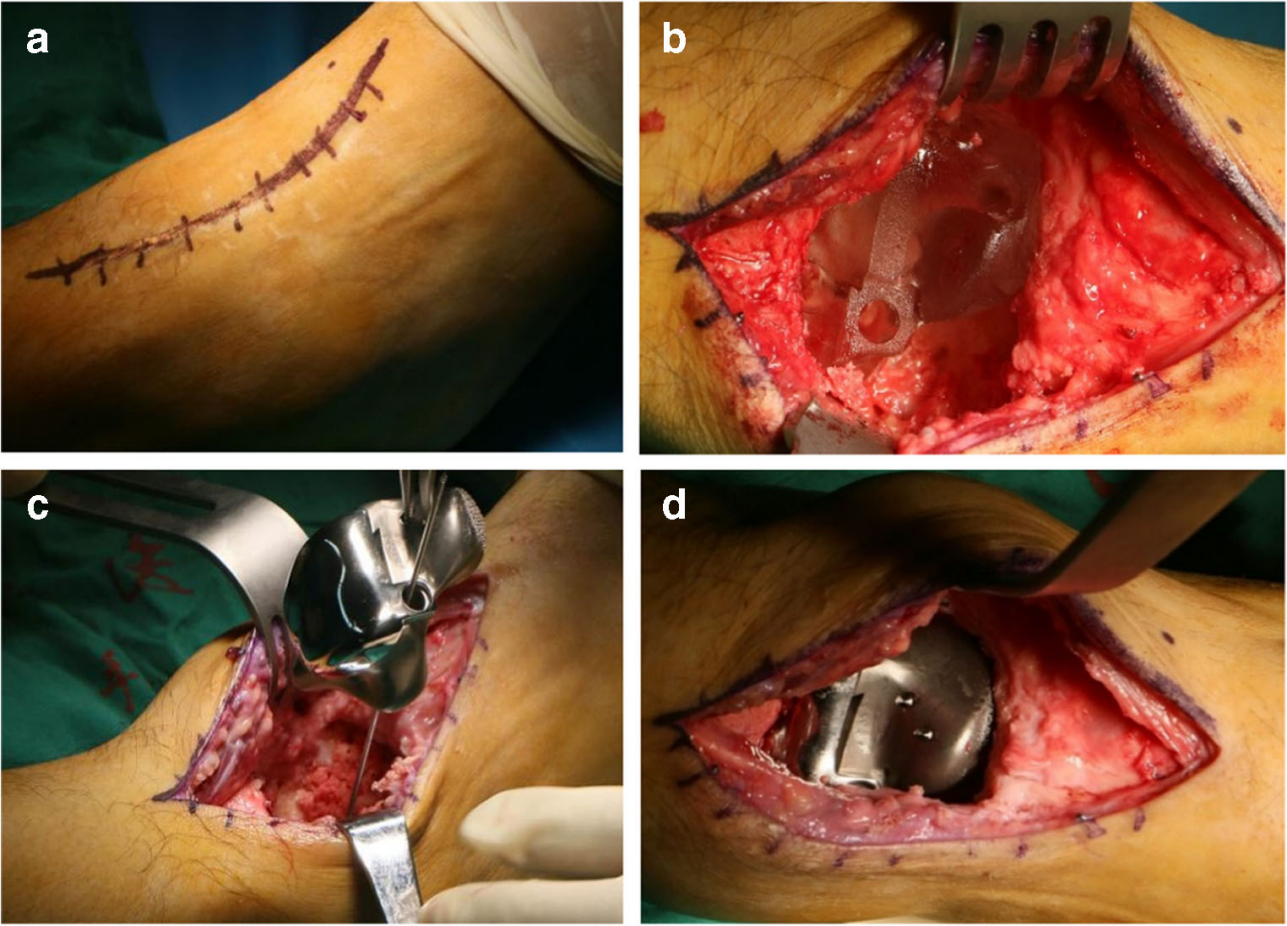
**a** A straight anteromedial incision approximately 12 cm in length was made in the right ankle. **b** The individualized prosthesis test model was inserted. **c** The individualized prosthesis was inserted via the preset slot in the calcaneus along the lateral guide wire. **d** The individualized prosthesis was fixed

After successful lumbar anesthesia, the patient was placed in a supine position, and the pressure of the tourniquet at the root of thigh was set as 300 mmHg. A straight anteromedial incision approximately 12 cm in length was made in the ankle to avoid injury to the dorsal nerve of the foot and anterior tibial nerve and vessels (Fig. 3a). Two 2.5-cm Kirschner wires were drilled into the anterolateral tibial ridge and cuboid, and then, a custom-made spring was placed; thereafter, the residual talus was removed, and the talonavicular and subtalar articular facets were resected to the subchondral bone. After extreme plantar flexion of the ankle joint, the prosthetic accuracy test model was inserted in an anterior-to-posterior direction, and then, the size of the test model and its degree of matching with the adjacent bone surfaces were observed and measured. Two slots (depth 1 cm) were made in the calcaneus via two preset holes in front of the prosthetic accuracy test model according to the preoperative design. Then, 1 guide wire was drilled into the lateral fixation hole in the individualized prosthetic test model. Finally, a hole was drilled in the calcaneus with a drill, and its depth was measured (Fig. 3b).

The prosthetic accuracy test model was removed and rinsed 3 times with a flusher. Bone morphogenetic protein (BMP) compound gel was uniformly spread on the calcaneal and navicular surfaces. Then, the base of the individualized prosthesis was inserted via the preset slot in the calcaneus along the lateral guide wire and beaten with a hammer to fit tightly with the calcaneus (Fig. 3c), and the lateral guide wire was withdrawn. After extreme plantar flexion of the ankle joint, the articular facet of the accurate prosthesis was inserted in an anterior-to-posterior direction via the slot in the surface of its base to ensure a tight fit.

The guide wire was drilled into the lateral hole in the test prosthesis again; a 6.5 mm cannulated screw with a proper length was inserted along the guide wire, the talonavicular and subtalar joints were placed in a neutral position, a 2.5-cm Kirschner wire was drilled into the lateral calcaneus, a 4.5 mm cannulated screw with a proper length was inserted along the guide wire, and then the individualized prosthesis was fixed (Fig. 3d). After the position, size, and matching of the talar prosthesis were confirmed by radiography, a partial short fibular muscle tendon was resected and then reconstructed on the anterolateral side of the prosthesis, and good movement of the ankle and good stability of the prosthesis were proven by examination. Thereafter, an indwelling negative-pressure drainage catheter was placed, and the incision was sutured layer by layer.

### Evaluation

**Fig. 4:**
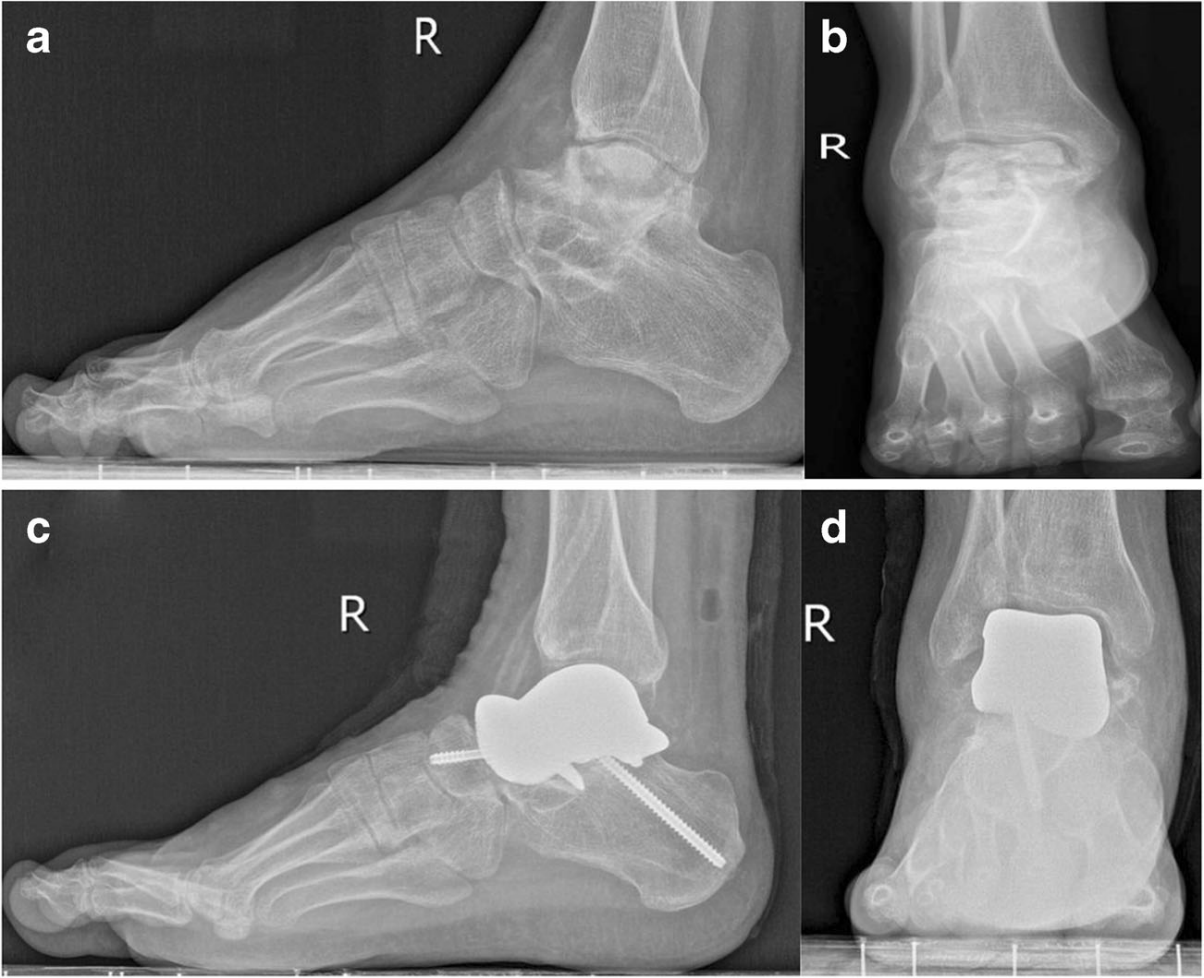
Lateral and anteroposterior X-rays of the right foot with complete TNC and the intact left foot were compared preoperatively (**a**, **b**) and postoperatively (**c**, **d**)

The patient was urgent to preserve ankle function after treatment. Functional exercises of dorsiflexion and flexion were performed to improve the range of ankle motion. The accuracy of the postoperative reconstruction with respect to the preoperative plan was evaluated in terms of the talar arc length, talar height, talar width, tibial alignment angle, talar tilt angle, Bohler’s angle, and Meary’s angle [17]. As well as strict follow-up from the first day after operation, imaging data were taken regularly and compared with those before operation (Fig. 4). The range of motion of the ankle, the AOFAS score, VAS score, the incidence of complications, and the satisfaction score regarding pain relief, activities of daily living, and return to recreational activities were recorded to evaluate ankle function recovery.

### Statistical analysis

The tested data conform to normal distribution and the paired-samples *t* test was performed for statistical analysis to compare the pre-operative and post-operative talar arc length, talar height, talar width, tibial alignment angle, talar tilt angle, Bohler’s angle, Meary’s angle, AOFAS score, and VAS score and the range of ankle motion using the Statistical Package for Social Sciences, version 13.0 (SPSS, INC., Chicago, IL, USA). All tests were two-tailed, and *P* <0.05 suggested a statistically significant difference. The 95% confidence interval (CI) of the difference was recorded.

## Results

No patient was lost to follow-up. The total operation time was 144.33 ± 48.30 minutes (range, 91~234 min), the blood loss volume was 63.33 ± 52.79 ml (range, 10~150 ml), and the length of hospital stay was 16.83 ± 5.27 days (range, 12~26 days). After a mean follow-up of 23.17 ± 6.65 months (range, 18~36 months), degenerative arthritis and prosthetic dislocation were not observed on plain radiographs. Each 3D-printed talar prosthesis was placed in the original anatomical position. On average, we observed bone fusion through X-rays, CT, and MRI at the interface occurred after 13.50 ± 1.76 weeks (range, 12~16 weeks).

### Imaging assessments

The pre-operative and post-operative talar arc length was 61.23 ± 6.33 mm (range, 52.36~67.54 mm) and 59.12 ± 4.15 mm (range, 54.40~64.82 mm) (95% CI − 1.89~6.12, *t* = 1.36, *P* = 0.233). The pre-operative talar height was 27.59 ± 5.99 mm (range, 19.00~27.60 mm), and the post-operative talar height was 34.56 ± 3.54 mm (range, 30.00~38.64 mm) (95% CI − 13.05 to − 0.87, *t* = 2.94, *P* = 0.032). The pre-operative talar width was 43.95 ± 6.40 mm (range, 37.41~53.31 mm), and the post-operative talar height was 41.96 ± 4.00 mm (range, 36.59~46.30 mm) (95% CI − 4.99~9.06, *t* = 0.74, *P* = 0.492). The pre-operative tibiotalar alignment angle was 84.83 ± 7.11° (range, 76.14°~92.15°). The post-operative tibiotalar alignment angle was 88.34 ± 4.50° (range, 80.41°~93.18°) (95% CI − 9.07~2.06, *t* = − 1.62, *P* = 0.166). The pre-operative talar tilt angle was 2.57 ± 3.25° (range, 0.04°~8.69°). The post-operative talar tilt angle was 1.10° ± 2.02° (range, 0.09°~5.22°) (95% CI − 0.31~3.24, *t* = 2.12, *P* = 0.087). The pre-operative and post-operative Bohler’s angle was 43.35 ± 10.56° (range, 31.13°~56.02°) and 37.75 ± 10.51° (range, 23.69°~53.69°) (95% CI − 2.47~13.69, *t* = 1.79, *P* = 0.134). The pre-operative Meary’s angle was 11.73 ± 4.79° (range, 7.32°–35.10°), and the post-operative Meary’s angle was 4.45 ± 1.82° (range, 0.09°–12.59°) (95% CI 1.29~22.44, *t* = 2.89, *P* = 0.034) (Table 2).

**Table 2:** Comparison of accuracy by radiographic parameters

	Before surgery	At the last visit	95% CI	*T* value	*P* value
Talar arc length (mm)	61.23 ± 6.33	59.12 ± 4.15	− 1.89~6.12	1.36	0.233
Talar height (mm)	27.59 ± 5.99	34.56 ± 3.54	− 13.05~-0.87	2.94	0. 032
Talar width (mm)	43.95 ± 6.40	41.96 ± 4.00	− 4.99~9.06	0.74	0. 492
Tibial alignment angle (°)	84.83 ± 7.11	88.34 ± 4.50	− 9.07~2.06	− 1.62	0. 166
Talar tilt angle (°)	2.57 ± 3.25	1.10° ± 2.02	− 0.31~3.24	2.12	0.087
Bohler’s angle (°)	43.35 ± 10.56	37.75 ±10.51	− 2.47~13.69	1.79	0.134
Meary’s angle (°)	11.73 ± 4.79	4.45 ± 1.82	1.29~22.44	2.89	0.034

### Functional outcome assessments

The average AOFAS score improved from an average of 26.33 ± 6.62 (range, 18~36) preoperatively to 79.67 ± 3.14 (range, 75~84) at the final follow-up (95% CI 43.36~63.30, *t* = 13.75, *P* = 0.000). The VAS score decreased from 6.33 ± 1.03 (range, 5~8) to 0.83 ± 0.75 (range, 0~2) (95% CI 4.40~6.60, *t* = 12.84, *P* = 0.000). The range of dorsiflexion improved from 7.56 ± 5.72 (range, 0~15) to14.22 ± 6.56 (range, 8~25) (95% CI − 12.43 to − 0.90, *t* = − 2.67, *P* = 0.029) (Table 3). The range of plantarflexion improved from 22.01 ± 9.86 (range, 3~30) to 29.22 ± 8.67 (range, 10~40) (95% CI 29.22 ± 8.67, *t* = − 8.95, *P* = 0.000) (Table 2).

**Table 3:** Comparison of clinical analysis

	Before surgery	At the last visit	95% CI	*T* value	*P* value
AOFAS	26.33 ± 6.62	79.67 ± 3.14	43.36~63.30	13.75	0.000
VAS	6.33 ±1.03	0.83 ± 0.75	− 13.05 to − 0.87	12.84	0.000
Dorsiflexion	7.56 ± 5.72	14.22 ± 6.56	− 12.43 to − 0.90	− 2.67	0.029
Plantarflexion	22.01 ± 9.86	29.22 ± 8.67	− 8.80 to − 5.20	− 8.95	0.000

### Complication assessments

All patients were able to do normal activities 1 month after surgery. Weakness and numbness of the foot and ankle were not observed. One patient complained of residual pain that he could endure. The postoperative satisfaction scores regarding pain relief, activities of daily living, and return to recreational activities were good to excellent in six cases.

## Discussion

In most cases, ankle replacement is contraindicated in TNC due to weight-bearing damage and excessive bone loss of the talus [12]. The common surgery for TNC are talectomy and arthrodesis of the four peritalar joints, but the former easily leads to peritalar instability, and the latter is prone to limiting ankle movement [18–20]. Therefore, reconstructing the talus and restoring ankle function are major challenges for orthopaedists [12].

Prosthetic replacement is a good approach for total talar reconstruction and functional ankle restoration. There have been only a few reports of talar prostheses in the literature [10, 21, 22]. In 1997, Harnroongroj et al. [23] reported that a satisfactory effect was observed in only 2/12 cases of prosthetic talar replacement, so widespread application was not feasible in clinical practice due to poor efficacy. The main cause may be that current talar prostheses have the following weaknesses: (1) several fixed models are produced in batches by manufacturers and thus fail to meet the differential demands of patients in terms of individual talar anatomy and biological functions; (2) the prostheses offer only structural support and has no biological functions. Therefore, designing and producing individualized talar prostheses with biological functions is a major issue in the preparation and clinical application of talar prostheses [18].

3D printing technology offers a new means of fabricating individualized talar prostheses and it is more suitable for manufacturing small batches of products with a complex shape and structure; thus, it shows strong promise for doctors to provide efficient, precise, medical solutions for patients [24, 25].

In the 3D printing design, we extracted and segmented the radiography, CT, and MRI scan data of healthy and affected taluses for precise reconstruction of the talar imaging data, reconstructed and matched the intact 3D raw data of the healthy and affected sides using mirror and data registration technology, established the articular facet model, and completed the smoothing and simplification processing [26]. Besides, we fitted the talar prosthesis model and the adjacent joints, established the fixation channel, and finished the virtual surgical simulation of total talar prosthesis implantation and fixation. In this way, complete matching of the talar prosthesis and the surgical operability of implantation were assured. Joseph Tracey reported no significant changes in the talar width and arc length in 14 patients who underwent the custom 3D-printed total talar prostheses replacement; however, the talar height was significantly increased, and Meary’s angle was decreased [17]. In this study, the talar height and Meary’s angle were significantly changed, which is similar to the literature. This means the talus hight is the most important dimension in designing prosthesis. Restoring the talus height can normalize the anatomical distribution between the hindfoot and forefoot.

In 3D printing production, this prosthesis has the following advantages: (1) all components of the 3D-printed structure were designed according to the functional anatomy of talus and Wolf’s law, which solves issues regarding the anatomical and biomechanical suitability of the prosthesis [27]. (2) This kind of prosthesis overcomes technical difficulties concerning the composite technology of three types of materials in the prosthesis, friction interface, and surface coating; strengthens the rigidity, tenacity, and fatigue resistance; and realizes the structural composition and high performance required. (3) The prosthetic tibial surface was made of cobalt-chromium-molybdenum alloy (Co-Cr-Mo), with a effective stiffness similar to that of the tibial plafond and showed enhanced performance as a tibiotalar prosthesis, including low friction, high wear degradation resistance, which maximize the restoration of joint movement function and the service life of the prostheses [16]. The lower surface material consisted of Ti6Al4V (Ti 6Al 4V), which can promote bone growth and increase stability. Moreover, there were two lower holes; the diameter of the through hole was 4.6 mm, and that of the screw was 4.5 mm, which was beneficial for the stability of the prosthesis. (4) The surface was fabricated by the microporous processing of the medial and posterior subtalar joint and talonavicular articular facet, and the hydroxyapatite coating and BMP compound gel were used to promote the rapid fusion and growth of the bone at the prosthetic interface.

We performed the surgical procedures according to the pre-operative surgical simulation. After resection of the residual talus, it is critical to maintain the matching of the talar prosthesis with the adjacent tibia, navicular, and calcaneus. We used a custom-made spring to maximize the spatial expansion of the talar implantation site and performed extreme plantar flexion of the ankle joint during implantation to ensure that there was sufficient space to implant the prosthesis. To guarantee the stability of the prosthesis, we fixed the interface of the prosthesis with the navicular and calcaneus separately with screws and slots and resected a partial short fibular muscle tendon to reconstruct the anterolateral side of the prosthesis, which can prevent detachment or displacement of the prosthesis after implantation. During the operation, we verified the stability and matching of the prosthesis by checking the negative movement of the ankle joint.

Considering the newly published study about 3D-printed custom talar prothesis by Rishin J. Kadakia [28, 29], this study is quite different. The concrete differences are as follows: (1) different casting methods of prostheses, each adjacent joint surface of the prosthesis has been specially treated; there is also the difference between the bioactive 3D-printed personalized talus prosthesis and the traditional 3D-printed talus prosthesis. (2) The inclusion criteria of this study are more stringent, only aiming at irreversible talus collapse and necrosis (that is, talus collapse and necrosis that cannot be solved by arthrodesis). (3) In order to strengthen the stability, the screws in the direction of inferior articular joint and navicular joint are designed in the casting process of prosthesis, unlike the way in which the talus prosthesis is not fixed with screws. We hypothesized that it is the reason why patients can recover ankle joint activity. In addition, unlike Scott’s [29] study, we only used the AOFAS score instead of FAOS score. Compared with AOFAS, FAOS pays more attention to the subjective feelings of patients. For the evaluation of the early efficacy of prosthesis, FAOS seems to be superior to AOFAS score. In the next included cases, we will collect data of AOFAS and FAOS at the same time to strengthen follow-up.

In conclusion, the individualized 3D-printed total talar prosthetic replacement has the following merits: (1) the prosthesis is designed according to variations in talar anatomy among patients and thus has complete anatomical suitability and meets the demand for accurate functional reconstruction. (2) The surgical safety and reliability are both improved so that orthopaedists can finish the prosthetic model design and surgical simulation pre-operatively and complete the complicated plan for total talar prosthetic replacement, thus reducing the operation time and ensuring surgical safety and operability. (3) The implanted 3D-printed talar prosthesis can achieve the maximum normal biological functions and promote bone growth/fusion between the calcaneus or navicular and the talar prosthesis through prosthetic surface/interface design by the use of BMP compound gel, thus restoring the normal anatomical structure and mechanical properties of the talus, shortening the recovery time and allowing patients to regain the normal biological functions of the ankle.

However, there are still some limitations that we cannot ignore: (1) the inclusion criteria of this study are too strict, resulting in fewer follow-up cases; (2) the biomechanical mechanism of specific surgical methods has not been studied, but the team is doing research in relevant aspects and will soon get results; (3) the follow-up time is too short to fully evaluate the stability and service life of a prosthesis. However, according to Taniguchi’s previous research [30], the service life of the talus prosthesis can reach an average of 52.8 months. We will continue to follow-up and continue to include patients who meet the inclusion criteria for treatment.
